# Enhancing the Stability of Aqueous Dispersions and Foams Comprising Cellulose Nanofibrils (CNF) with CaCO_3_ Particles

**DOI:** 10.3390/nano8090651

**Published:** 2018-08-23

**Authors:** Tiia-Maria Tenhunen, Tiina Pöhler, Annaleena Kokko, Hannes Orelma, Michel Schenker, Patrick Gane, Tekla Tammelin

**Affiliations:** 1 VTT Technical Research Centre of Finland Ltd., P.O. Box 1000, FI-02044 VTT Espoo, Finland; tiia-maria.tenhunen@vtt.fi (T.-M.T.); tiina.pohler@vtt.fi (T.P.); annaleena.kokko@kemira.com (A.K.); hannes.orelma@vtt.fi (H.O.); 2 Omya International AG, Baslerstrase 42, CH-4665 Otringen, Switzerland; michel.schenker@fiberlean.com; 3 FiberLean Technologies, Par Moor Centre, Par Moor Road, Par, Cornwall PL24 2SQ, UK; 4 Department of Bioproducts and Biosystems, School of Chemical Engineering, Aalto University, FI-00076 Aalto, Helsinki, Finland

**Keywords:** cellulose nanofibrils (CNF), hybrid hydrocolloid pigments, percolation network, dispersion stability, cellulose-based foam, plateau border stability in aqueous foams, inorganic-organic hybrid materials, nanocellulose-CaCO_3_ containing foams, stability enhancement of foams

## Abstract

In this work, stability of dispersions and foams containing CaCO_3_-based pigments and cellulose nanofibrils (CNF) was evaluated with the aim to reveal the mechanisms contributing to the overall stability of the selected systems. The utmost interest lies in the recently developed hydrocolloid hybrid CaCO_3_ pigments and their potential to form bionanocomposite structures when incorporated with CNF. These pigments possess a polyelectrolyte layer deposited on the surface of the particle which is expected to enhance the compatibility between inorganic and organic components. Stability assessment of both dispersions and foams was conducted using turbidity profile scanning. In dispersions, CNF provides stability due to its ability to form a firm percolation network. If surface-modified pigments are introduced, the favourable surface interactions between the pigments and CNF positively influence the stability behaviour and even large macro-size pigments do not interfere with the stability of either dispersions or foams. In foams, the stability can be enhanced due to the synergistic actions brought by CNF and particles with suitable size, shape and wetting characteristics resulting in a condition where the stability mechanism is defined by the formation of a continuous plateau border incorporating a CNF network which is able to trap the inorganic particles uniformly.

## 1. Introduction

Hybrid structures comprising nanoscale cellulosic materials and inorganic minerals are an important material combination for diversity of applications varying from, for example, biomedical devices to renewable packaging materials and fire-retardant nanocomposites [1,2,3,4,5,6]. Furthermore, the bionanomaterial architectures with high porosity and large surface area combined with renewable and sustainable material attributes have gained increasing interest to be used, amongst other things, as high efficiency air filters and as substrates for biocatalytic conversion [7,8]. In the realm of porous bionanocomposite architectures, the vital element to be developed is the integration of viable manufacturing technologies ensuring the controlled construction of strong, light-weighted functional structures with economically feasible routes. Here, the foam-forming technology brings several crucial features with respect to both improved material performance and potential savings in terms of raw materials, energy and water [9,10]. Recently Al-Quararah et al. (2015) showed that using aqueous foam-forming technology the microporosity of the cellulose fibre network can be tailored by the bubble-size distribution, which was seen to have a direct influence on the final mechanical performance of the formed networks [11].

The unique properties of cellulose nanofibrils (CNF) such as high aspect ratio, large interfacial area, excellent hydrogen bonding and gel forming ability, coupled with high strength and amphiphilic character, make them an auspicious candidate to be exploited in many technical applications [12] as well as building blocks in advanced materials [13].

Mineral filler particles are often used to enhance the stiffness, rigidity and impact strength of polymeric composite materials. Recently, Dimic-Misic et al. (2016) introduced an approach involving the inclusion of in situ CaCO_3_ precipitation in order to prepare uniform nanocellulose-based inorganic-organic composites [14]. To achieve enhanced solid nanocomposite properties derived from the mineral inclusion, it is necessary to ensure full dispersion of both constituents, nanocellulose component and inorganic component, and to induce dispersion compatibility between the nanofibrils and the inorganic particles. The control over the interactions between CNF and inorganic particles is indeed a key defining factor determining the structural uniformity and final physical performance of all kinds of systems whether it is a dilute aqueous dispersion, wet foam or solid porous nanocomposite structure. More recently, Liu et al. (2017), showed how, by suppressing Brownian motion through inducing sufficient stress within the gel-like network, calcium carbonate nanoparticles could be adsorbed onto nanofibrils, revealing the proton donating nature of the trapped water at the fibril surface [15].

Previously, it has been shown that surface xylan located on nanofibrils plays a significant role as an electrosteric stabilizer in dilute cellulose nanofiber (CNF) dispersions when the surface forces are dominant whereas the removal of surface xylan drastically changes the CNF dispersion stability and alters the rheological behaviour of the fibrils [16,17,18]. The settling of the unstable CNF dispersions displays behaviour which is typical for hindered sedimentation [16]. In addition, in more concentrated systems cellulose nanofibres enhance the dispersion stability by creating a percolation network through the dispersion structure [16,17]. When pigments are added to CNF dispersion, low surface charge may easily lead to flocculation, whilst surface modification of the CNF fibrils with anionic CMC and the pigment with cationic polyelectrolyte, respectively, improves the hybrid dispersion stability [19]. Schenker et al. (2016a and 2016b), illustrated also that micro nanofibrillated cellulose dispersion stability could be enhanced by addition of CMC during manufacture, and, furthermore, coprocessing with CMC-treated calcium carbonate pigment could lead to enhanced strength-giving properties in compact dried composites [20,21]. These findings were subsequently developed to enable paper coatings to be designed with significantly reduced amounts of micro nanocellulose used as binder, due to enhanced fibrillar CMC-treated pigment interaction [22]. 

Particles in a foam can act either as an antifoaming agent or conversely as a stabiliser. The effect depends on the interfacial tensions between the air–water–particle phases. Surface-active particles have been used as stabilisers in industrial applications for decades. In practice, particles can stabilise foams if they are not too hydrophilic, such that the contact angle between water and the particle surface is not too small [23]. Cellulose is an amphiphilic polymer, and utilisation of amphiphilic particles has been popular for preventing or delaying the separation of phases and, thus, in the making of stable foams [24]. Previously, we have demonstrated that similar CNF grade with high xylan content is able to efficiently stabilise water-in-oil emulsions against coalescence via Pickering mechanism [25]. The mechanism behind the stabilising effect of CNF in wet foams has been previously studied by Cervin et al. (2015) [26]. According to that study, the stabilising effect of CNF in wet CNF foams is based on the formation of a fibril network generating an associated gelling effect, in turn immobilising water at the air–water interface. 

The question posed in this work is whether the percolation network and the gelling behaviour of CNF can be utilised as a stabiliser in dispersions and foams that contain either micro (nanometre)size or macro (micrometre)size CaCO_3_-based pigments. The investigations also include the optimisation steps in respect to pH and ionic strength levels, as well as pigment surface charge control and hydrogen bonding potential provided by a deposited polyelectrolyte layer on the topmost surface of the pigment particles. In addition, the stability of the generated foam systems was studied using two surface active agents, a traditional foaming agent sodium dodecyl sulfate (SDS) and polymeric polyvinyl alcohol (PVOH). We attempt to reveal the mechanisms contributing to the enhanced overall stability of the selected systems with the aim ultimately to construct foam-templated porous bionanomaterial hybrid structures. 

## 2. Materials and Methods

### 2.1. Cellulose Nanofibrils (CNF)

Once-dried elemental chlorine-free (ECF) bleached birch Kraft pulp from Finnish pulp mill (Metsä Fibre, Äänekoski, Finland) was used as a raw material for production of cellulose nanofibrils (CNF). The chemical composition of the pulp was 72.7% cellulose, 25.6% xylan, 0.6% glucomannan, 1.0% lignin and 0.1% acetone extracted substances [27,28,29]. To control the fibre swelling, and to enhance the fibrillation, the carboxyl groups of the pulp were converted to the sodium counter ion form prior to the fibrillation according to the method described previously [30,31] with some exceptions [32]. Briefly, the metal counter ions were removed by adjusting the pulp pH below 3 with 0.01 M HCl. After filtration and washing with deionised water, re-conversion of the carboxyl groups into their sodium form was achieved by mixing the pulp in 0.005 M NaHCO_3_ solution. The pH was subsequently adjusted to 8–9 with 1 M NaOH and, finally, the pulp was washed with deionised water until the conductivity of the filtrate was below 20 µS cm^−1^. The anionic charge of the washed pulp was 39 mmol kg^−1^ and the chemical composition analysed for parity with the starting material once again as 72.7% cellulose, 25.6% xylan, 0.6% glucomannan, 1.0% lignin and 0.1% acetone extracted substances [27,28,29].

**Figure 1:**
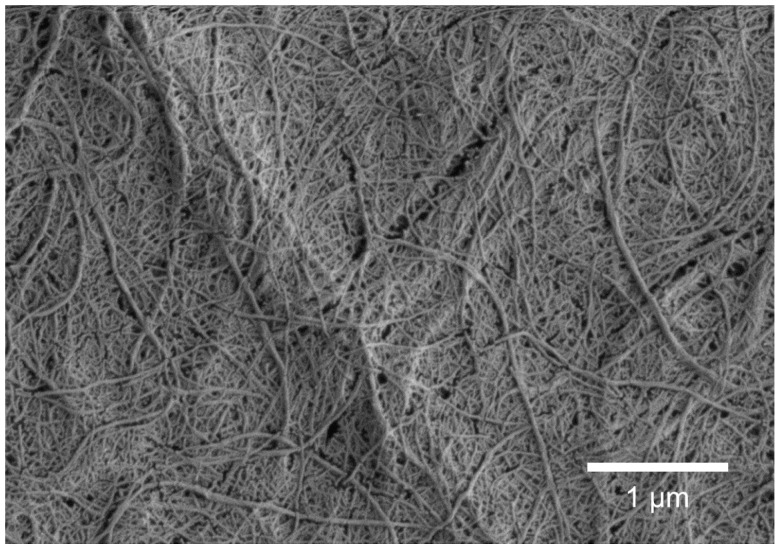
SEM image of cellulose nanofibrils (CNF) mechanically disintegrated from bleached birch Kraft pulp showing fibrils with the width of 50–500 nm and the length of several micrometres.

Cellulose nanofibrils (CNF) were produced by means of mechanical disintegration: the pulp was first soaked at 1.7 w/w% consistency and dispersed using a high shear Diaf dissolver (Diaf Pilvad Aps, Fredensborg, Denmark) for 10 min at 700 min^−1^ (rpm). Then pulp pre-refining was carried out with an ultrafine friction grinder (MKZA10-15J, Masuko Sangyo Co., Ltd., Kawaguchi-City, Japan) at 1500 min^−1^. Thereafter, CNF fibrillation was performed using a high-pressure fluidiser (Microfluidiser, M7115-30, Microfluidics Corp., Newton, MA, USA). The pulp was first passed once through a chamber pair having respective diameters 500 µm and 200 µm, followed by five passes through a second chamber pair of 500 µm and 100 µm diameter. The operating pressure was 1850 bar. The final appearance of CNF was a viscous gel with a solids content of 1.6 w/w%, at pH 6.7, having a conductivity of 45 µS cm^−1^. Scanning electron microscopy (SEM) imaging of CNF was carried out with a Merlin Field Emission (FE)-SEM (Carl Zeiss NTS GmbH, Oberkochen, Germany). The electron gun voltage was set to a constant 3 kV with a grid current of 60 pA. The pixel resolution was 2048 × 1536. The final fine structure of the high aspect ratio fibrils is shown in Figure 1 and Table 1 lists the basic properties of CNF with respect to solids content, pH and conductivity. In addition, the total charge states of the CNF dispersions (ζ-potentials), determined using a Zetasizer Nano ZS equipment (Malvern Instruments Ltd., Malvern, UK), are collected in Table 2.

### 2.2. Pigments

**Figure 2:**
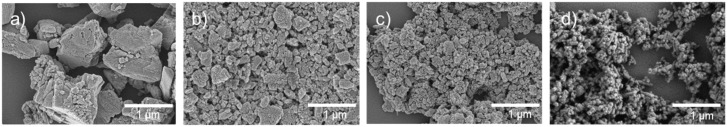
SEM images of the CaCO_3_-based pigments (**a**) PHCH; (**b**) nanoPHCH0; (**c**) nanoPHCH1; (**d**) nanoGCC.

Based on the benefits seen in respect to generating improved dispersibility and compatibility together with increased binding power between calcium carbonate and CNF [20,21,22], the CaCO_3_ used were pre-treated in production with polysaccharide. This is exemplified here by carboxymethyl cellulose (CMC, M_w_ ~250 kg mol^−1^, degree of substitution (DS) 0.7, CP Kelko, Äänekoski, Finland), termed pigment hydrocolloid hybrid (PHCH). Three such different fine ground calcium carbonate-based (PHCH) slurries were provided by Omya International AG (Otringen, Switzerland) and used as received. The range chosen exhibited, respectively, a comparison between a micrometre (PHCH-termed macro, using the standard scientific nomenclature) and two nanosized (nanoPHCH0 and nanoPHCH1-termed micro, also for nomenclature consistency) materials. The microsized grades represented two different surface charge levels, namely strongly anionic and weakly anionic, the latter being achieved by partially neutralising the highly charged surface adsorbed CMC using a cationic polyelectrolyte, poly(diallyldimethylammonium chloride) solution (pDADMAC, M_w_ < 100 kg mol^−1^, Sigma-Aldrich, Saint Louis, MO, USA). A nanosized CaCO_3_ grade without polyelectrolyte pretreatment (nanoGCC) was also utilised in dispersion stability investigations as a reference material. SEM images of the pigments are presented in Figure 2 and their basic properties with respect to structure, size and charge in Table 3.

### 2.3. SEM Imaging of The CNF and Pigments

Scanning electron microscopy (SEM) imaging including cellulose nanofibrils and pigments was carried out with a Merlin Field Emission (FE)-SEM (Carl Zeiss NTS GmbH, Oberkochen, Germany). The electron gun voltage was a constant 1.5 kV with a grid current of 60 pA. The pixel resolution was 2048 × 1536. The SEM specimens were prepared by further dilution of the previously diluted CNF (0.016 w/w%) and pigment dispersions (0.01–0.02 w/w%) with MilliQ-water (1:1 dilution, Merck Millipore, Burlington, MA, USA). Then droplets of given solutions were applied on pure silicon dioxide wafers, followed by evaporation of water under ambient conditions. Prior to the SEM imaging, the dried specimen surfaces were gold sputter-coated (30 mA, 30 s).

### 2.4. Other Chemicals

To avoid pH fluctuations, all the dispersions and foams were prepared using an aqueous tris base buffer solution (NH_2_C(CH_2_OH)_3_, Trizma^®^ base, M_w_= 121.14 g mol^−1^, Sigma-Aldrich, Saint Louis, MO, USA). pH of the dispersions was adjusted using 0.1 M or 0.5 M HCl (Sigma-Aldrich, Saint Louis, MO, USA). For foam experiments sodium dodecyl sulphate (SDS, 99%, Sigma-Aldrich, Saint Louis, MO, USA) and polyvinyl alcohol, PVOH (Poval 6–88, degree of hydrolysis 86.7–88.7 and M_w_ of 37,000, Kuraray Europe GmbH, Hattersheim, Germany) were used as foaming agents. All the chemicals were laboratory grade and used as received.

### 2.5. Preparation of Dilute Pure CNF Dispersions and Pigment-Containing CNF Dispersions

For the stability assessment, aqueous dispersions with varying concentrations of CNF (0.025 w/w% and 0.05 w/w%) as well as pigment-containing CNF dispersions with varying pigment: CNF ratios (4:1, 1:1, 1:4, total concentration *c*_tot_ = 0.05 w/w%) were prepared using a high-speed mixer (Ultra Turrax T25, IKA Works GmbH, Staufen, Germany). Dispersions were prepared using two levels of ionic strength (5 and 10 mM) and two pH levels (pH 8 and pH 8.5). First, the pigment slurries were diluted to concentrations of 0.01–0.02 w/w% and CNF to 0.016 w/w%, respectively. 30 g samples of CNF or CNF-pigment dispersions were prepared by adding well-dispersed diluted pigment dispersion, diluted CNF, buffer solution (either 1.5 g or 3 g depending on the ionic strength) and deionised water. The dispersion was mixed for 1 min at 25,000 min^−1^ and the dispersion stability was analysed directly after mixing. 

### 2.6. Preparation of CNF-stabilised Pigment-Containing Foams

Single material reference foams (pure surfactant foams, pure CNF foams with surfactants and pure pigment foams with surfactants) and compound CNF stabilised pigment-containing foams were prepared by adding surfactants (0.6 g dm^−3^ SDS or 2 g dm^−3^ PVOH) and then mixing the dispersions with the high-speed mixer. Dispersions were buffered to avoid any pH fluctuations (pH 8 and ionic strength of 5 mM). All the components (CNF, pigments, buffer, water and surfactant) with total volume 60 cm^3^ were added in a glass decanter and mixed for 1 min at 7000 min^−1^ and 2 min at 24,000 min^−1^. The air content of the generated foam (final foam volume) was measured immediately after the mixing step. Air content was calculated based on the initial volume of the solution and the final volume of the foam. The foam was instantaneously inserted into a Turbiscan glass cell for the foam stability measurement. 

### 2.7. Stability of Dilute CNF Dispersions and Pigment-Containing CNF Dispersions

The stability of dilute CNF dispersions without and with pigments was measured using a Turbiscan turbidity analyser (Turbiscan LAB, Formulaction SA, L’Union, France), in which the transmission and backscattering of light are measured. A method is based on the Faraday-Tyndall effect and the ability of colloidal systems to scatter light. The CNF dispersions, and pigment-containing CNF dispersions were analysed in a cylindrical glass cell geometry in scanning mode, where the optical reading head scanned the length (height) of the sample cell (up to 55 mm), acquiring light transmission data every 40 µm, equivalent to the illumination cross-section size. Data were collected every 5 min for 1 h. The measurements were plotted as curves over time, where transmitted light flux values are provided in percentages relative to standards (suspension of monodisperse spheres and silicone oil) as a function of the sample height, measured on millimetres. 

### 2.8. Stability of CNF Stabilised Foams and Pigment-Containing CNF Foams

The stability of foams was investigated using vertical turbidity profile scanning (Turbiscan turbidity analyser). The refractive indices of air and dispersion are sufficiently different such that they create contrast in the intensities of transmission and backscattering. The intensity of transmitted and backscattered light, respectively, therefore depends and the distribution of air and constituent components in the dispersion, such that the relative transmitted intensity throughout a scan of a given foam depends on the amount of air in its pathway. 

The stability of reference foams (pure surfactant foams, pure CNF with surfactants and pure pigments with surfactants) as well as pigment-containing CNF foams was measured instantaneously after the foam was formed. The transmission signal was recorded to analyse the turbidity of the drained phase of the foam in the bottom of the glass cell. The stability of the foam was assessed by following the backscattering data as a function of time similarly as described above for transmitted light flux. Changes in backscattering signal reveals the bubble growth as a function of time. Bubble growth can be detected as a drop in the backscattering curve due to bubble coalescence. Foam stability data were collected every 1 min for 10 min.

## 3. Results and Discussion

### 3.1. Assessment of Dispersion Stability

Stability of both dilute CNF and pigment-containing CNF dispersions (prior to foaming) as a function of dispersion concentration was assessed by measuring the light transmission profiles as a function of time. In addition, the influence of pH and ionic strength on the dispersion behaviour was analysed.

#### 3.1.1. Stability of Dilute CNF Dispersions

**Figure 3:**
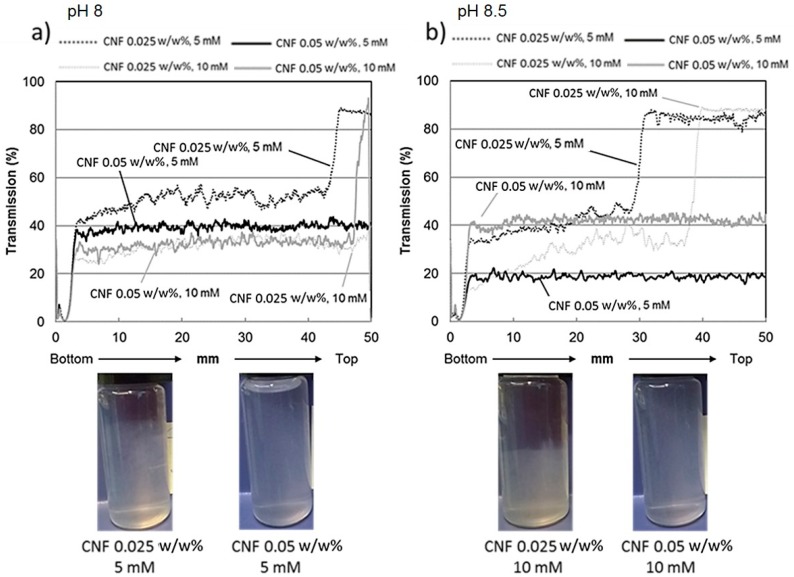
Transmission profiles and photographs of dilute CNF dispersions at (**a**) pH 8 and (**b**) pH 8.5. Samples are scanned from bottom to top, *t* = 1 h.

Figure 3 shows the light transmission curves along the sample cell for pure dilute CNF dispersions with fibril concentration of 0.025 w/w% and 0.05 w/w%, respectively. The light transmission data were collected at pH levels of 8 and 8.5 and ionic strength levels of 5 mM and 10 mM to reveal how sensitive the system was towards surface electrostatic-induced changes. 

As shown by the Figure 3, all the 0.05 w/w% CNF dispersions display transmission curves showing no variations along the whole cell height. This indicates that no sedimentation of the fibrils is taking place over the defined time scale of 1 h. Thus, we may conclude that the fibrils are stable against agglomeration. CNF dispersions with lower concentration (0.025 w/w%) seem, however, to be more susceptible towards agglomeration. Cellulose nanofibrils settle to some extent, seen as clear variations in turbidity along the cell height. However, neither pH nor electrolyte change induces any severe sedimentation under these experimental conditions. Photographs presented in Figure 3 give further visual information on the appearance of the selected dispersion after 1 h, and confirm the scan results. 

Based on the light transmission profiles and the visual appearance of the sample cells, the 0.05 w/w% CNF dispersion seems to be the most stable one. This is fully in accordance with our previous findings with a similar CNF dispersion [16]. The mechanisms determining the stability behaviour arise from three different sources. The surface of the fibril is covered by loosely bound and anionically charged glucuronoxylan, which efficiently stabilises dispersions via both electrostatic and steric interactions, collectively termed electrosteric stabilisation [33]. The birch Kraft pulp-based CNF grade used in this work contains 25% of xylan, which is a source for weak repulsive forces between the fibrils. The total charge of the CNF dispersions evaluated via ζ-potential measurements (Table 2) is varying between −17 mV and −25 mV regardless of the pH or ionic strength. These values indicate that the electrostatic contribution to the stability is low, and relatively small changes will interfere with the stability in the absence of other stabilising mechanisms. The third mechanism contributing, or even dominating stability behaviour, is the percolation network formed by the fibrils. In the percolation network, the individual fibrils form entanglements at low solids content forming a gel-like network structure throughout the dispersion phase. This network contributes to the system stability due to the formation of viscous gel, which leads to collective frameworks of fibrils preventing them from migration, and thus inhibiting severe agglomeration and associated complete sedimentation. 

CNF dispersion with lower concentration (0.025 w/w%) settles until the individual fibrils reach a high enough state of compaction under which they no longer move. At this state the local viscosity is high enough that the strong percolation network is again formed, see also the photographs in Figure 3. Previously, we have demonstrated similar settling behaviour, called hindered sedimentation, for CNF dispersions with low xylan content (lower anionic charge and less mobile xylan molecules located on the fibril surface), see [16]. The CNF dispersion in this case seems to be somewhat less stable compared to the work by Tenhunen et al. (2016), probably due to the presence of slightly coarser CNF utilised in this work. Here, the mechanical disintegration of fibrils from pulp fibres was far less intensive, leading to lower viscosity and a broader size distribution of the formed fibrils. All these features have influence on the overall colloidal stability of the system. 

#### 3.1.2. Stability of Pigment-Containing CNF Dispersions

**Figure 4:**
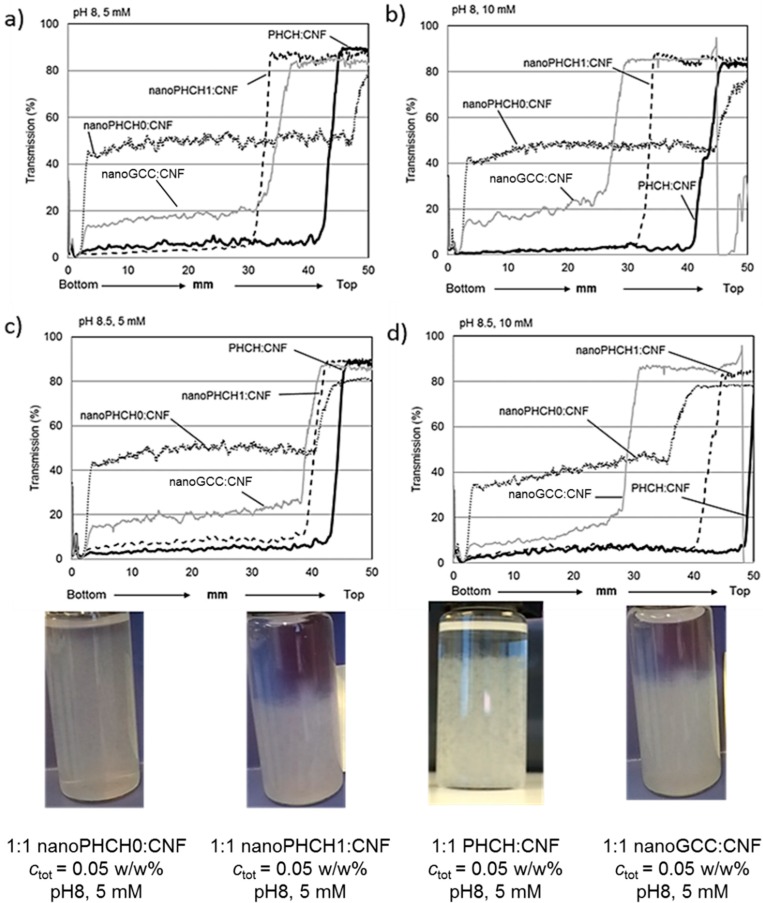
Transmission profiles of pigment-containing CNF dispersions with the concentration ratio of 1:1, *c*_tot_ = 0.05 w/w%: (**a**) pH 8 and ionic strength 5 mM; (**b**) pH 8 and ionic strength 10 mM; (**c**) pH 8.5 and ionic strength 5 mM and (**d**) pH 8.5 and ionic strength 10 mM. Samples are scanned from bottom to top, *t* = 1 h. Photographs show the visual appearance of the selected dispersions after 1 h.

Figure 4 shows the light transmission profiles along the sample cell for four different pigment-containing CNF dispersions determined after 1 h. The concentration ratio of pigments and cellulose nanofibrils is 1:1 with a total concentration of 0.05 w/w%. The light transmission data were again collected at pH levels of 8 and 8.5 and ionic strength levels of 5 mM and 10 mM. 

The nano-scaled hydrocolloid hybrid pigment (nanoPHCH0)-containing CNF dispersions showed the smallest light transmission variations, and the stability behaviour was more or less independent of the pH and ionic strength. This particular calcium carbonate-based nanosize pigment carries relatively high anionic surface charge of −711 µVal g^−1^, determined by polyelectrolyte titration. The origin of the surface charge is derived from the carboxymethyl cellulose (CMC) molecules adsorbed on the inorganic pigment surface. Colloidal forces can be expected to play the key role: high repulsion forces between anionically charged and nanosize particles (both pigments and CNF) efficiently stabilise the dispersion, even at such low CNF concentration such that the CNF-based percolation network is not yet fully formed (0.025 w/w%). The surfaces of both nanoparticle species contain also a mobile polymeric component (CMC on pigment and xylan on CNF), which can also be a source for steric repulsive forces, thereby hindering any severe agglomeration and sedimentation and simultaneously decreasing the sensitivity towards electrostatic-induced changes.

The CNF dispersions that contain the other nanoscale hydrocolloid hybrid pigment (nanoPHCH1) seem to be more susceptible towards agglomeration, although complete sedimentation behaviour cannot be detected, see Figure 4. Minor changes in pH rather than changes in ionic strength seem to affect the stability behaviour. At pH 8 the agglomeration and settling tendency seem to be more pronounced compared to when at the slightly higher pH 8.5. This hybrid pigment contains two oppositely charged polymeric molecules, anionic CMC and cationic polyDADMAC, attached on the calcium carbonate surface, resulting in the surface charge of −599 µVal g^−1^, see Table 3. This pigment, therefore, carries to some extent a lower surface charge value when compared to the highly anionic nanoPHCH0 pigment. Therefore, the electrostatic repulsion between the nanosize components in the CNF dispersion can be expected to be lower leading to higher susceptibility towards agglomeration and settling. Complete sedimentation of the dispersion can be avoided due to the percolation network that is formed once the dispersion is sufficiently settled, see also the respective photograph in Figure 4. In this case the percolation network derived stability and hindered sedimentation are the main mechanisms which define the dispersion behaviour.

The comparison of the behaviour of the nanosize hybrid pigments to nanosize CaCO_3_ grade (nanoGCC) without polyelectrolyte treatment reveals that all the nanoGCC dispersions are relatively unstable. Regardless of the pH and ionic strength levels the shape of the light transmission curves is similar, showing that the nanoGCC dispersions gradually settle, although once again complete sedimentation is not taking place due to the formation of a percolation network formed by CNF, see also the photograph of the vial of nanoGCC containing CNF dispersion. 

The behaviour of the dispersion system that contains macroscopic hydrocolloid hybrid pigment, PHCH, is somewhat unexpected, see the light transmission curves in Figure 4 and the corresponding photograph. After one hour, regardless of the utilised pH or ion strength levels, no severe settling can be detected. The turbidity level of CNF dispersions containing macro-sized PHCH is clearly higher when compared to the dispersions with nano-scaled pigments, since the PHCH pigment size is almost ten times larger, see Table 3. Simultaneously, the surface charge and surface area are significantly lower and therefore the tendency of the dispersions to agglomerate and settle might be anticipated. However, it seems that CNF and PHCH particles interact in such a way that the CNF network is strong enough to carry even the large macroscale pigments. Also, in this case, we consider that the main mechanism which defines the dispersion stability is the firmness of the percolation network, but the importance of the favourable surface interactions between fibrils and pigment particles cannot be ruled out. Indeed, the comparison to the reference system of nanoGCC, that lacks both the better compatibility with CNF as well as steric hindrance generated by the incorporated polyelectrolyte layer, supports this interpretation. Nanosized CaCO_3_ containing dispersion with the similar surface charge level (see Table 3) is clearly agglomerating whereas CaCO_3_-based hybridized PHCH dispersion acts to maintain sufficient stability.

**Figure 5:**
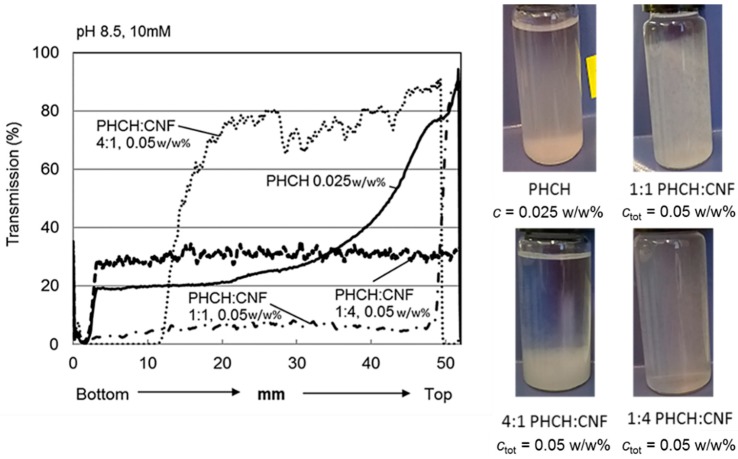
Transmission profiles of macroscopic pigment (PHCH)-containing CNF dispersions with different concentration ratios, *c*_tot_ = 0.05 w/w% at pH 8.5 and ionic strength of 10 mM. Samples are scanned from bottom to top, *t* = 1 h. Photographs show the visual appearance of the dispersions after 1 h.

The ability of the CNF network to support the macro-sized PHCH pigment was further evaluated by varying the ratio of cellulosic nanofibrils and pigments. The experimental conditions selected for the comparison (pH 8.5 and ionic strength 10 mM) were those which resulted in the most stable dispersions. Figure 5 shows the light transmission profiles of macroscopic pigment-containing CNF dispersion with the concentration ratios of 1:4, 1:1 and 4:1 (pigment:CNF) with the corresponding photographs of the vials after the turbidity scan (1 h). For comparison purposes, also the transmission profile of pure PHCH dispersion is shown. It is evident that the CNF network structure traps the pigment particles and stabilises the dispersion. Dispersions with concentration ratios of 1:1 and 1:4, respectively, yield profiles with no changes along the vial height. Dispersions with the weight concentration ratio of 4:1 (0.04 w/w% pigment and 0.01 w/w% CNF) settle completely during 1 h. In all cases the profiles are completely different when compared to the profile of pure PHCH pigment. 

### 3.2. Assessment of Foam Stability

The stability of foams was investigated using turbidity profile scanning using the Turbiscan. The refractive indices of air and dispersion are sufficiently different such that they create observable contrast in the intensities of transmission and backscattering. The transmitted and backscattered light intensities depend on the amount of air in the illuminated pathway. During destabilisation of the foam, the amount and placement of air in the structure change within the measuring cell due to coalescence of the bubbles accumulating from the top of the sample in the vial and liquid drainage toward the bottom. These effects can be monitored as changes in both the transmission and backscattering signals. The smaller the bubbles, the higher is the backscattering intensity. If the concentration of the dispersed air bubbles is high enough, the transmission signal will be extinguished, since the light becomes so highly scattered that it does not pass the sample. The transmission signal observed from the top of the measuring cell indicates the shift of the foam meniscus. If a transmission signal is observed on the bottom simultaneously as the backscattering signal, the signal is a secondary light reflection and must be ignored. Foam ripening can be observed as a parallel shift of the backscattering curve over time to lower intensity values [34]. 

#### 3.2.1. Stability of Reference Foams

**Figure 6:**
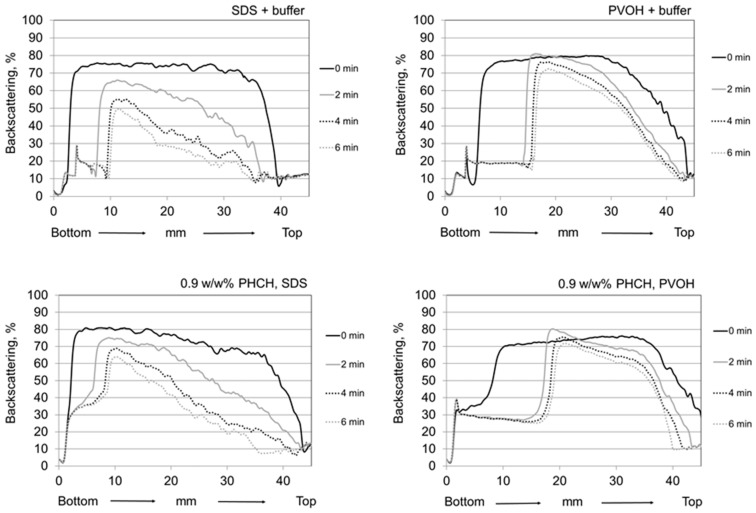
Backscattering profiles of pure foaming agents (top row) and pure macroscopic pigment PHCH, *c* = 0.9 w/w% at pH 8.5 and ionic strength of 5 mM. Foam samples are scanned from bottom to top at time intervals of 0, 2, 4 and 6 min after foaming.

**Figure 7:**
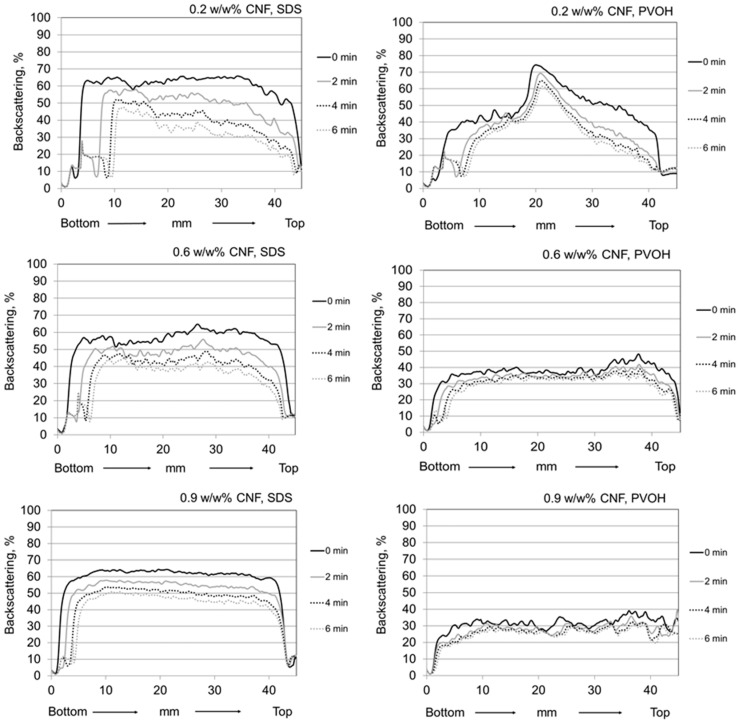
Backscattering profiles for pure CNF containing foams with foaming agents and with varying CNF concentration at pH 8.5 and ionic strength of 5 mM. Foam samples are scanned from bottom to top at set time intervals up to 6 min.

According to the findings of the pigment-containing CNF dispersion stability investigations, the selected conditions for the foaming experiments were pH 8.5 and ionic strength of 5 mM. Figure 6 and Figure 7 show the backscattering curves along the sample cell for selected reference systems, i.e., for both pure foaming agents (anionic SDS and amphoteric polyvinyl alcohol (PVOH)) and for pure macroscopic hybrid pigment (PHCH) containing foams with foaming agents (Figure 6), and for pure CNF containing foams with foaming agents and with varying CNF concentration (Figure 7) recorded at selected times of 0, 2, 4 and 6 min after foam formation.

The backscattering data in Figure 6 show that pure PVOH foam had greater stability in the time frame of 10 min when compared to the SDS foam, seen as a smaller drop of backscattering profile as a function of time. In addition the bubble size was slightly smaller in PVOH foam than SDS foam, which could be deduced from multiple backscattering intensity measurements showing higher intensity from the PVOH derived foam. Both foam systems demonstrated fast drainage. The addition of macroscale pigment did not significantly change the behaviour of the foam system. More stable foam was achieved with polymeric PVOH and there was no significant difference in the bubble size. In addition, the observably fast drainage took place in both systems composed of pigments and foaming agents together. 

Figure 7 shows the stability behaviour of CNF containing foams having three different concentrations of nanofibrils, 0.2 w/w%, 0.6 w/w% and 0.9 w/w%. By increasing the CNF concentration, the foam air content is seen to decrease, whilst the foam stability increases, as viewed over the defined time scale of 6 min, and simultaneously the drainage is reduced. It is also rather evident that CNF foam with PVOH as a foaming agent displays higher stability when compared with the SDS containing system. Additionally, the air content of CNF foams with PVOH is lower than it is with the corresponding ones prepared using SDS. The collected backscattering curves indicate that the highest stability was achieved using 0.9 w/w% nanocellulose. As discussed by Cervin et al. (2015), the stabilising effect of CNF foams is based on the formation of the percolation network and gel-formation at the water/CNF-air interface [26]. Due to this combination of mechanisms, the complex viscoelastic modulus of the air–water interface can be considered to increase, which has a positive influence on the foam stability.

Polyvinyl alcohol as a foaming agent represents a polymeric surface-active chemical, and the desired foaming ability of the PVOH with a similar degree of hydrolysis (DH) in the presence of CNF has been previously demonstrated by Hou and Wang (2017) [35]. PVOH is not only efficiently generating CNF containing foams but can also improve bonding strength of individual fibrils [36]. In addition, both of these materials are hydrophilic with amphiphilic character. Such materials are considered to improve the foam stability since they can accumulate in foam plateau borders and lamellae, and further irreversibly adsorb at the air–water interface [37]. The hydrated CNF-PVOH network located within the plateau borders and lamellae can be expected to reduce drainage due to high viscosity within the lamella, in turn preventing gas diffusion from bubbles and bringing structural reinforcement and resistance against foam coarsening, all positive factors when considering enhanced foam stability [37,38,39]. 

#### 3.2.2. Stability of Pigment-Containing CNF Foams

**Figure 8:**
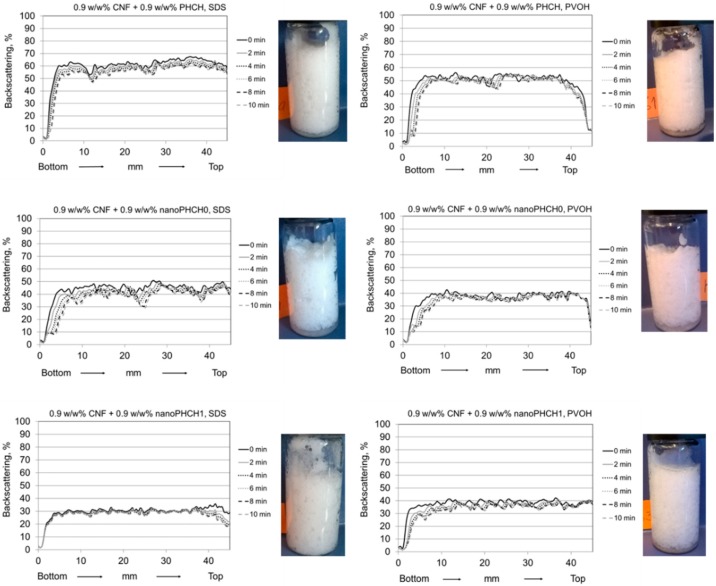
Backscattering profiles from pigment-containing CNF foams with the concentration ratio of 1:1, *c*_tot_ = 1.8 w/w% at pH 8.5 and ionic strength of 5 mM. Samples are scanned from bottom to top for 10 min. Photographs show the visual appearance of the foams immediately after the scans.

The selection of CNF-pigment systems for the foam stability experiments was based on the findings achieved in the CNF-pigment dispersion stability evaluations. The most stable dispersions were achieved with nanosize and macrosize hybrid pigments (nanoPHCH0, nanoPHCH1 and PHCH), see Figure 4. The dosage levels of the constituent components were selected according to the foam stability investigations of the reference systems. Dispersions containing 0.9 w/w% of CNF result in foams with the highest stability with both surface-active agents, see Figure 7. Therefore, pigment-containing CNF dispersions with the concentration ratio of 1:1 (0.9 w/w% CNF + 0.9 w/w% pigment) were selected for the foam experiments. These conditions ensured the CNF network structure was able to efficiently trap the pigment particles efficiently, and thus is strong enough to carry even the largest macroscale particles. Finally, the stability of pigment-containing CNF foams as a function of time using the two different foaming agents (anionic SDS and amphoteric polyvinyl alcohol (PVOH)) was assessed by measuring the backscattering profiles and visually examining the corresponding photographs of the vials after the backscattering scan, see Figure 8. Due to the excellent stability observed at the original time setting of 6 min, a further additional 8 and 10 min time lapse steps were added, i.e. stability was retained at least 10 min after initial foaming, and this is confirmed visually from the photographic images taken after scanning.

It is apparent that the introduction of hybrid pigments to the CNF dispersion leads to the formation of foams with significantly improved stability. All of the formulations resulted in stable foams regardless of the addition of pigment particles. The remarkable stability improvement was observed for SDS foams, and seen as completely unaltered backscattering profile during the scanning period of 10 min. For the reference, all SDS foams without pigments displayed clear changes in the backscattering profile, see Figure 6 and Figure 7. PVOH-containing foams were also highly stable within the time scale of 10 min and the bubble size seems to be finer, seen as a higher intensity level when compared to foams without pigments.

**Figure 9:**
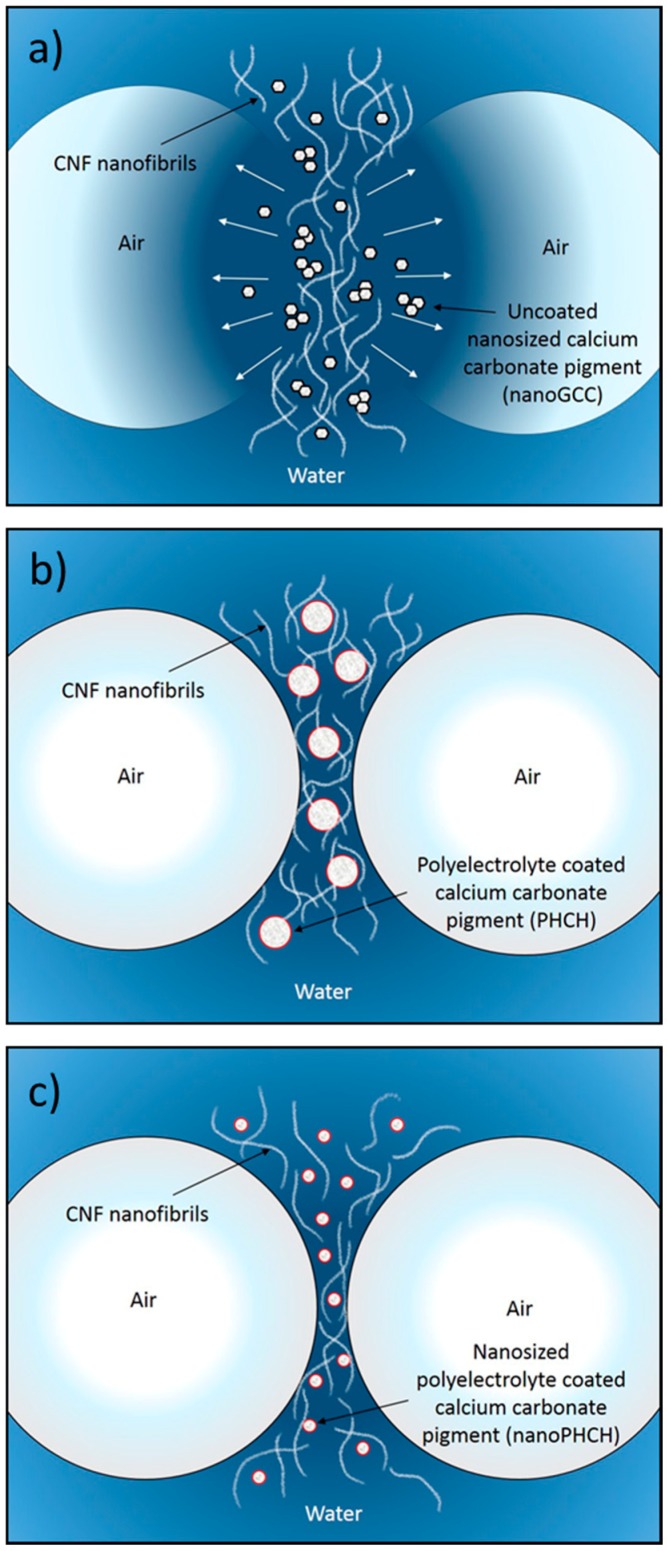
Schematic representations of unstable and stable aqueous foam systems containing CNF and pigments where the stability mechanism is proposed to be due to the formation of continuous plateau border and percolation network. (**a**) Incompatibility between CaCO_3_ and CNF leads to the flocculation of the components resulting in discontinuous plateau border and lamella rupturing; (**b**) and (**c**) The favourable interactions between hybridised CaCO_3_-based pigments and CNF provides plateau border continuity, and the foam stability is efficiently maintained due to the percolation network formed by CNF. Even the macro-scaled pigments do not interfere and break the lamella.

Synergistic actions brought by cellulose nanofibrils and inorganic particles with suitable size, shape and wetting characteristics can be expected to be a source for the efficient foam stabilisation. Therefore, the foam stabilisation mechanisms are multifaceted. As discussed above, the percolation network of high aspect ratio CNF increases the viscosity of lamellae, bringing reinforcement due to the high number of contact points between entangled fibrils, and simply increases resistance against foam coarsening. On the other hand, the stabilisation mechanism of inorganic particles with low aspect ratio has been stated to derive from the adsorption of the particles at a bubble interface. The adsorbed layer is then able to suppress the disproportionation (gas diffusion during Ostwald ripening), thus preventing bubble coalescence. This type of behaviour has been previously reported, for example, for surface modified oxide particles [39] and surface modified rod-like CaCO_3_ particles [40]. In our case, an additional interaction must be considered, which is related to the continuity of the liquid phase in the foam lamellae between the air bubbles in the presence of fibrils and inorganic particles. Favourable interactions between hydrated CNF and hybridised pigments (also in a hydrated form) can be expected to result in an even distribution and suitable location of both particles in lamellae and plateau borders, which further ensures the continuity of water throughout the liquid phase in the foam system. The schematics of the proposed structure of a foam lamella in the presence of CNF and pigment particles is depicted in Figure 9.

The favourable interactions between CNF and pigments can be derived from the CNF-pigment dispersion stability results, see Figure 4 and Figure 5. The agglomeration and sedimentation of the dispersion systems are efficiently hindered once the dispersions are stabilised via electrosteric mechanisms and, as a result, the firm percolation network is formed. This is indeed the case when hydrocolloid hybrid pigments are utilised along with cellulose nanofibrils, and this applies even with macrosize pigment (PHCH). Despite the surfactant action of lowering the surface tension of the water medium, the suspended particles remain in the liquid phase probably due to their appropriate surface wetting characteristics. Both particle species carry mobile and hydrophilic polymeric components and chemical groups on their surface which increase the surface hydration. Hydrocolloid hybrid pigments together with CNF are aligned along the lamellae and they are expected to provide adequate inter-bubble lamellar thickness, see Figure 9. If, however, the particle size of some of the component particles exceeds the inter-bubble lamellar thickness, and is not surface treated, whilst other particles can be housed within it, the large particle acts to distort the lamella, thus increasing the surface area contact between water and air, which then destroys the metastable equilibrium and the bubble will collapse. Our findings are in accordance with Kadoi and Nakae (2011) who demonstrated how the distribution and location of Al_2_O_3_ and SiC particles depend on the energy balance at the solid-liquid interface and how the energy balance is influencing the formation of continuity between liquid and solid in foam systems [41]. It can be concluded that CNF acts as a structural skeleton in CNF-pigment foams due to gel formation and the intertwined gel-forming network. The gel-like CNF suspension is able to carry the same or smaller amount, in terms of mass, of surface modified pigment particles with favourable interactions without compromising the stability of the foam. Despite the short measurement time reported here, it was possible to observe that the age of the most stable foam phases spanned 24 h.

## 4. Conclusions

The percolation network and gel formation of CNF in dilute dispersions and foams is effective enough to carry up to the same or smaller mass of pigment without compromising the stability, if the pigment–CNF interactions are favourable. In some cases, the stability can even be improved due to the synergistic effects brought by the system where CNF and pigment particles form a continuous and firm network with high compatibility, which can be achieved either by controlling pigment particle size or further enhanced by adopting a surface-treated calcium carbonate pigment, in the form of a polymer hydrocolloid hybrid, in this case exemplified by the polysaccharide carboxymethyl cellulose. These findings serve as a route indicator to construct true nano-enhanced composite structures in a controlled manner, simultaneously avoiding the agglomeration and uneven distribution of the individual components. 

## Figures and Tables

**Table 1:** Basic properties of CNF.

Dry solids content (w/w%)	1.6
pH of CNF gel	6.7
Conductivity of CNF gel (µS cm^−1^)	45
Conductivity of 0.05 w/w% CNF dispersion (µS cm^−1^)	25
Conductivity of 0.05 w/w% CNF dispersion, I = 5 mM (µS cm^−1^)	465 at pH 8.0 370 at pH 8.5

**Table 2:** ζ-potential values of 0.05 w/w% buffered CNF dispersions as a function of pH and ionic strength.

pH and Ionic Strengt	ζ-Potential
pH 8.0, 5 mM (mV)	−21.2 ± 0.2
pH 8.0, 10 mM (mV)	−17.2 ± 0.2
pH 8.5, 5 mM (mV)	−23.5 ± 1.1
pH 8.5, 10 mM (mV)	−22.6 ± 1.8

**Table 3:** Composition, particle size, specific surface area and polyelectrolyte titrated charge (PET) of the CaCO_3_-based pigments.

	PHCH	nanoPHCH0	nanoPHCH1	nanoGCC
Incorporated polyelectrolytes	1.5 w/w% CMC + 0.6 w/w% pDADMAC	13 w/w% CMC	13 w/w% CMC + 5 w/w% pDADMAC	-
Median volume equivalent spherical diameter *d*_50_ (µm) *	1.5 (by weight)	0.14 (by number) 0.18 (by weight)	0.08 (by number) 0.15 (by weight)	0.11 (by number) 0.17 (by weight)
Specific surface area by BET (m^2^ g^−1^)	4	16 *	12 *	32
Charge by PET (µVal g^−1^)	−54	−711	−599	−34

* The measurement of such fine particles is not trivial as (re-)agglomeration is very strong. Therefore, the reported values must be considered approximate. However, the nanosize pigments are confirmed to be significantly smaller compared to the macroscale micrometre-size PHCH.
